# A case of angioid streaks that produced choroidal neovascularization after the onset of unilateral acute retinopathy in pseudoxanthoma elasticum

**DOI:** 10.1016/j.ajoc.2022.101591

**Published:** 2022-05-19

**Authors:** Akika Kyo, Manabu Yamamoto, Shigeru Honda

**Affiliations:** Department of Ophthalmology and Visual Sciences, Osaka Metropolitan University Graduate School of Medicine, Osaka, Japan

**Keywords:** Angioid streaks, Retinal pigment epithelium, Intraocular inflammation, White dots, Choroidal neovascularization

## Abstract

**Purpose:**

To report a case of angioid streaks that showed non-exudative choroidal neovascularization (CNV) after the onset of acute retinopathy in pseudoxanthoma elasticum (PXE).

**Observations:**

A 64-year-old woman with PXE visited our department for an ophthalmologic evaluation. Her decimal best-corrected visual acuity (BCVA) was 1.5, with angioid streaks (AS) around the optic disc in either eye at the first visit. Seven years later, her left eye's BCVA suddenly decreased by 0.3, and the fundus showed blurring of the Ellipsoid zone and vitreous cells along with the retinal pigment streaks on the nasal fovea. Diagnosed as acute retinopathy in PXE, twenty-two weeks after the start of oral prednisolone, the Ellipsoid zone became clear and the BCVA improved to 1.2, but CNV gradually developed. After intravitreal injection of bevacizumab, the CNV was decreased.

**Conclusions and importance:**

CNV on the AS lesion may occur after acute retinopathy in PXE.

## 1 Introduction

Pseudoxanthoma Elasticum (PXE) is a hereditary disease characterized by degeneration of elastic fibers and calcification. Ophthalmologically, retinal pigment streaks (Angioid Streaks, AS) due to breaks of Bruch's membrane and granular pigment abnormalities called Peau d'orange appearance are observed, and 50–70% of AS were reported to cause choroidal neovascularization (CNV). On the other hand, acute retinopathy may occur in PXE, which shows clinical manifestation similar to multiple evanescent white dot syndrome (MEWDS).^1^

We report a case of unilateral acute retinopathy that occurred during the follow-up period of AS in PXE patients, which subsequently developed CNV.

## 2 Case report

**Fig. 1:**
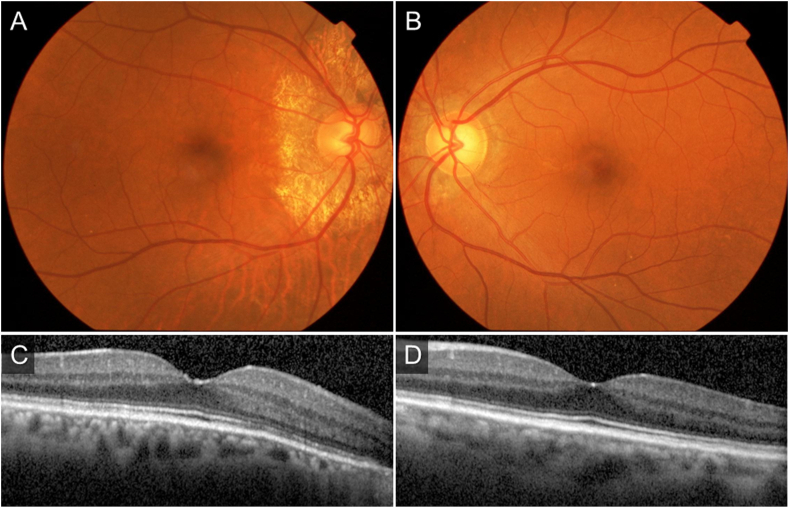
Findings of color fundus photographs and optical coherence tomography (OCT) at the first visit to our department. A, B: Color fundus photographs revealed angioid streaks around the optic disc in both eyes. C, D: OCT showed no particular abnormality.

A 64-year-old woman was diagnosed with PXE at the Department of Dermatology, Osaka City University, and referred to our department for the purpose of ocular examination. Decimal best-corrected visual acuity (BCVA) at the first visit was 1.5 with a Landolt C chart in each eye. AS around the papilla was observed in both eyes (Fig. 1) and a central choroidal thickness (CCT) at her first visit was 264 μm in the left eye and 170μm in the right eye. She was then followed-up every six months and the AS around the papilla was gradually expanded, but the macula was normal and no CNV was observed. The CCT gradually decreased with age, and CCTs became 163 μm in the left eye and 81μm in the right eye at 6 years and 3 months after the initial visit.

**Fig. 2:**
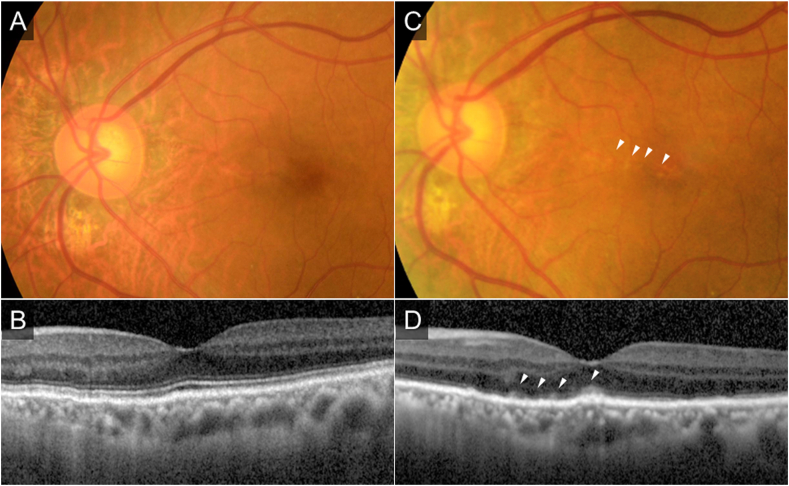
A, B: Clinical findings at 6 years and 3 months from the first visit. C, D: Clinical findings at 6years and 7months from the first visit (the time of onset). At the time of onset, a color fundus photograph in the left eye showed parafoveal granular white lesions in the deep layer of the retina (arrowhead) (C), and optical coherence tomography (OCT) showed blurring of the Ellipsoid zone and high-intensity lesions in the outer retina corresponding to the fundus white lesions (arrowhead) (D).

**Fig. 3:**
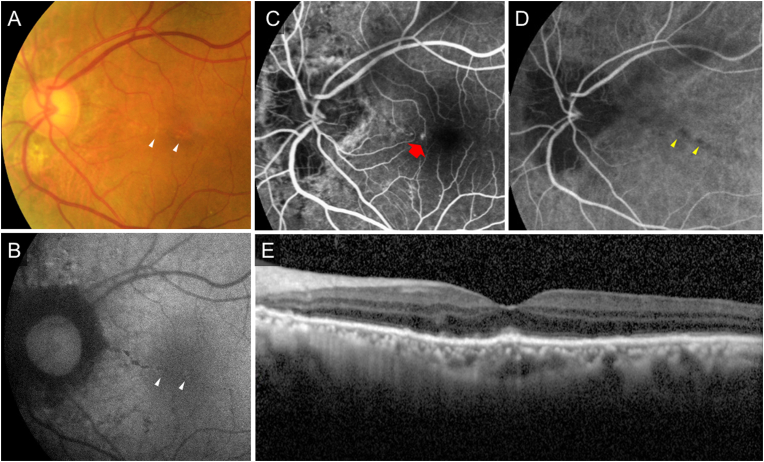
Findings of the fundus in the left eye at the onset of acute retinopathy. A: Color fundus photograph B: Fundus autofluorescence (AF) C, D: The early phase of fluorescein angiography (FA) and the late phase of indocyanine-green angiography (IA). E: Optical coherence tomography (OCT) AF showed hyperfluorescence consistent with fundus white granular lesions (white arrowhead) (B). FA showed a dot leakage (arrows) from the angioid streaks (AS) lesion in the nasal fovea (C). IA showed hypofluorescence along with AS (arrowheads) and a filling delay within the vascular arcade (yellow arrowhead) (D). Since fundus white lesions are present along with the AS, the hypofluorescence of the white lesions appears to be masked by that of the AS and is not noticeable. (For interpretation of the references to color in this figure legend, the reader is referred to the Web version of this article.)

Four months later, she visited our hospital with symptoms of vision loss and color vision abnormality in the left eye three weeks before the consultation. BCVA in her left eye decreased from 1.2 at the last visit to 0.3. There were no abnormalities in the anterior segment of the eye, while the left eye showed vitreous cells and parafoveal granular white lesions in the deep layer of the retina. Optical coherence tomography (OCT) showed blurring of the Ellipsoid zone (EZ) and high-intensity lesions in the outer retina corresponding to the fundus white lesions (Fig. 2). The CCTs were 199 μm in the left eye and 81μm in the right eye at this time point. Of these lesions, areas of particularly high brightness on OCT were hyperfluorescent on fundus autofluorescence (AF) (Fig. 3B). The fluorescein angiography revealed a dot leakage from the AS lesion in the nasal fovea and the late-phase indocyanine green angiography showed hypofluorescence along with AS and a filling delay around the AS lesion (Fig. 3C and D). Chest X-rays and blood tests were performed for differential diagnosis including uveitis, but no notable abnormalities were found.

**Fig. 4:**
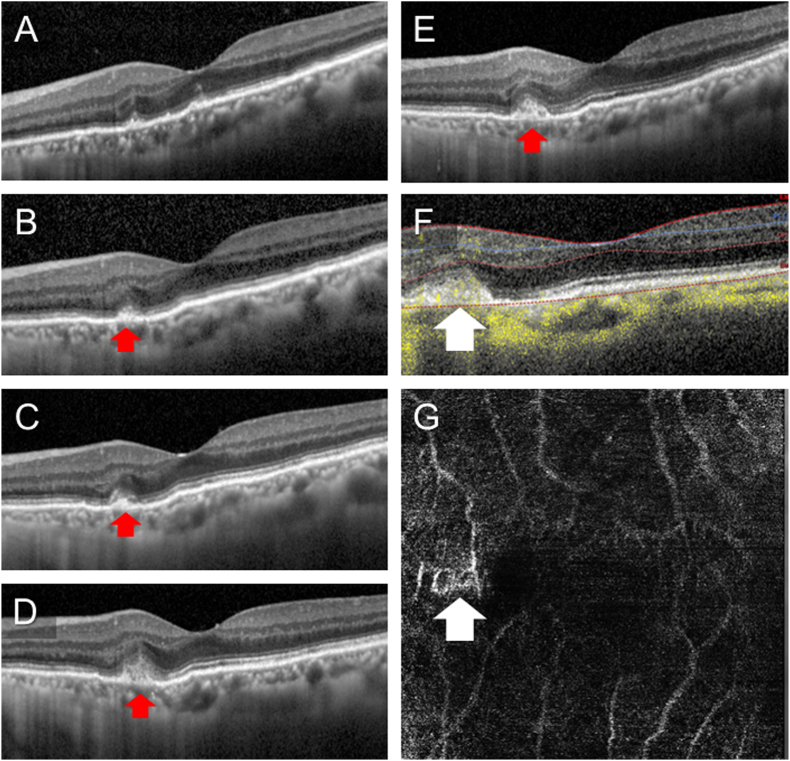
Chronological changes in the findings of optical coherence tomography (OCT) in the left eye. A: At the onset of acute retinopathy, B–E: At 5, 10, 17, and 22 weeks after starting oral prednisolone, respectively, F, G: B-scan image and en face image of OCT angiography at 22 weeks after starting oral prednisolone. The Ellipsoid zone became clear after the initiation of oral prednisolone while choroidal neovascularization gradually developed (arrows) and OCT angiography revealed the blood flow (big white arrow).

Based on the above, acute retinal pigment epithelitis was suspected. For treatment, 20 mg of oral prednisolone was started and the blurring of EZ was improved in 2 weeks. The dose was gradually reduced by 5 mg every 4 weeks after the start of prednisolone. The fundus white lesions and vitreous cells had disappeared, the EZ became almost clear at 22 weeks after the start of treatment, and BCVA in the left eye was improved to 1.2. The CCTs at week 22 were 149 μm in the left eye and 80μm in the right eye. There were no exudative changes during this time period such as subretinal fluid or intraretinal fluid, but CNV gradually developed on the AS lesion in the nasal fovea (Fig. 4). Then, an intravitreal injection of bevacizumab was administrated and CNV was regressed a month later with BCVA kept 1.2.

## 3 Discussion

As an ophthalmic abnormality associated with PXE, AS is reported to eventually develop in most patients, and it is well known that the risk of CNV development increases with age.^2^ On the other hand, Gliem et al. reported that acute retinopathy was found in about 5% of PXE as a relatively rare complication.^1^ They mentioned that acute retinopathy in PXE suggests inflammation in the retinal outer layer and shares some similarities with MEWDS; more common in women, unilateral, rate of vision loss, mild vitreous cells, ophthalmoscopic findings (multiple white dot lesions in the outer retina at the posterior pole), and findings in multimodal imaging. However, unlike typical MEWDS, acute retinopathy in PXE tends to show fundus lesions beneath AS lesions, retinal vessels, and optic disc. Since the outer retinal abnormalities were found along AS in the present case, we considered that they were pathological conditions associated with PXE rather than MEWDS.

Focusing on the CCT, as Hidalgo-Díaz et al. reported that the choroid of the PXE eye was thicker than that normal eye,^3^ the left CCT in this case was 264μm at the first visit which was thicker than normal.^4^, ^5^, ^6^ Her left CCT gradually decreased from the first visit to 4 months before the onset (163 μm) probably due to aging, but the CCT increased at the onset of acute retinopathy (199 μm) and decreased again after the remission. It eventually decreased to 149 μm 22 weeks after the onset of acute retinopathy. On the other hand, the CCT of the fellow eye showed almost no change after the onset of acute retinopathy in the left eye. In MEWDS, the choroid tends to increase in the acute phase and decrease in the recovery phase.^7^ This suggests that acute retinopathy in PXE may show a transient thickening of the choroid similarly to MEWDS. It is currently unclear whether choroidal thickening is more frequent in acute retinopathy in PXE than in MEWDS.

Gliem et al. further noted that acute retinopathy in PXE caused vision loss in 67% of cases, and central subretinal hyperreflective proliferations affected visual prognosis. In the present case, although there was a clear decrease in visual acuity at the onset, central subretinal hyperreflective proliferations were not observed, which was likely the reason why visual acuity at the final visit was improved. Central subretinal hyperreflective proliferations seen in MEWDS are thought as an accumulation of destroyed photoreceptor outer segment,^8^^,^^9^ Hence, the severity of inflammation in the outer retina might be associated with the appearance of central subretinal hyperreflective proliferations in the acute retinopathy in PXE.

Intravitreal injections of anti-VEGF agents, oral steroids, and triamcinolone injections have been used alone or in combination as treatments for acute retinopathy in PXE, but there is no consensus to date. The phenotypic similarity between acute retinopathy in PXE and MEWDS suggests that an autoimmune mechanism may be associated with the etiology of acute retinopathy in PXE similarly to MEWDS. Actually, autoantibodies have been reported in acute retinopathy in PXE.^1^ Although autoantibodies were not measured in this case, we hypothesized that some intraocular inflammatory mechanism or autoimmune mechanism may be involved in this condition, since the patient's abnormal fundus findings and symptoms improved after systemic steroid administration. However, a previous report described that anti-retinal and anti-retinal pigment epithelial antibodies were produced as a result of retinal degeneration, not a cause.^10^, ^11^, ^12^ Since there are too few reports to disclose the etiology of PXE, further investigations will be needed.

The frequency of non-exudative macular neovascularization was reported to be 33.3% in AS.^13^ The present case showed non-exudative CNV after the onset of acute retinopathy, which suggested that CNV was more likely formed due to inflammatory changes rather than the result of an expansion of AS lesion.

In conclusion, we experienced a case of unilateral acute retinopathy during the follow-up period of AS associated with PXE, and CNV developed concurrently with an improvement of acute retinopathy. Although there is still a lot to elucidate the mechanism of this clinical entity, it should be noted that CNV may occur after acute retinopathy in PXE.

## Funding

No funding or grant support.

## Authorship

All authors attest that they meet the current ICMJE criteria for Authorship.

## Declaration of competing interest

The following authors have no financial disclosures: (AK, MY, SH).

## Patient consent

Written consent was obtained from the patient.
